# Quantitative detection of THz-ATR spectra of aqueous samples under strong-field terahertz wave

**DOI:** 10.1016/j.isci.2022.105871

**Published:** 2022-12-24

**Authors:** Wei Shi, Chunhui Li, Haiqing Wang, Zhiquan Wang, Lei Yang

**Affiliations:** 1 Key Laboratory of Ultrafast Photoelectric Technology and Terahertz Science in Shaanxi, Xi’an University of Technology, Xi’an 710048, China

**Keywords:** Nanomaterials, Radiation physics

## Abstract

Owing to the characteristics of THz wave, the terahertz time-domain spectral (THz-TDS) system has potentials in the field of biological macromolecule detection. However, water with strong absorption effect on THz wave exists in most biological detection, so the research focus in this field is to study aqueous samples. In view of these, THz spectroscopy system has research value for qualitative and quantitative detection of α-lactose and its water-containing samples. This research used a THz-TDS system with LiNbO_3_ crystal to generate strong THz wave that was used to test 0.29 mmol α-lactose samples with water content of 15 μL–930 μL by using attenuating total reflection (ATR) prism. The absorption peak at 0.53 THz is detected, and with the increase of water content, the curve of absorption spectrum is observed to move up on the whole. This research has a guiding role for the test and improvement of water content limit in this field.

## Highlights

• THz-ATR spectra of α-lactose were obtained by LiNbO_3_ crystal THz-TDS system
• With increment of water in the sample, the absorption baseline is rising
• Detection limit corresponding to the water addition reaches 930 μL

## Graphical abstract

**Figure:**
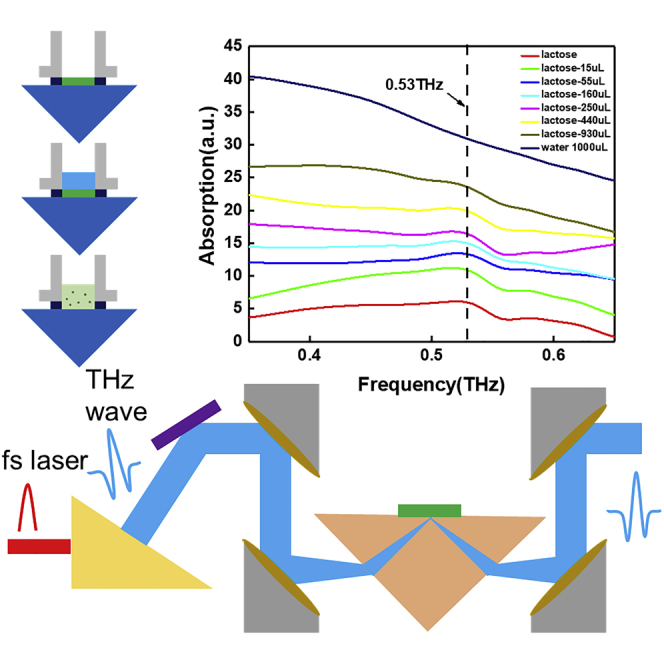

## Introduction

Terahertz wave is a kind of electromagnetic wave whose frequency is in the range of 0.1–10 THz. Its position in the electromagnetic spectral band is between millimeter wave and infrared wave, which happens to be in the transition stage of electronics and photonics. THz wave is characterized by low energy, high time resolution, strong penetration, and fingerprint characteristics. It can be applied to nondestructive testing based on its low energy characteristics.^1^ THz time-domain spectra with high signal to noise ratio and detection sensitivity can be obtained based on its high time resolution. Owing to its fingerprint characteristics, the photon energy of THz waves is on the same order of magnitude as the rotational and vibrational energy levels of most biological macromolecules, which means that the terahertz detection technique can detect the absorption peaks of these biological macromolecules. Therefore, the terahertz time-domain spectral system designed based on the combination of infrared spectral analysis system and THz wave is also suitable for the detection of biological macromolecules.

At present, there are mainly several detection methods in the field of aqueous sample detection, such as sample cell,^2^^,^^3^^,^^4^ attenuated total reflection technology (ATR), and microfluidic chip.^5^^,^^6^ Among them, ATR, which is widely used in infrared spectrum research, is mainly used to overcome the problems that are difficult to be solved by transmission detection method. Already there has been some research on detection of NaCl crystal,^7^ milk,^8^ glucose solution,^9^^,^^10^ rat’s neuron,^11^ human brain tumor tissues,^12^^,^^13^ skin tissue,^14^ and leaves.^15^ Compared with the traditional transmission spectra method, the ATR detection method has two features. Firstly, transmission spectra method requires more complex pretreatment of samples when compared with ATR. This method requires the penetration of the sample, and the absorbance of the sample to be tested needs to be taken into account, so the sample needs to be processed quite thin in part of the test, which has high technical requirements and also brings high costs, such as the detection method using microfluidic chip. In general, ATR does not need to do too much preprocessing for the test sample or even directly smear on the ATR prism. This method is simpler for sample preparation and replacement and can achieve *in situ* detection and reduce operational errors. Secondly, ATR generally uses optical sensor material, which can be reflected inside the sensor as long as it has a high enough refractive index. The design is based on the principle of total reflection of light, and the sample to be measured is placed on the surface of the ATR prism for detection using evanescent wave.

Currently, the THz sources commonly used in the field of photonics include photoconductive antenna and optical rectification mode. Compared with widely used photoconductive antennas, the optical rectification mode based on LiNbO_3_ crystal has an average THz pulse power 8 times that of the photoconductive antenna with similar energy conversion efficiency.^16^^,^^17^ However, due to the limited spectrum width of this system, it has not been widely used. Therefore, we preferentially selected α-lactose with an absorption peak at 0.53 THz as the sample to be tested. Lactose is a disaccharide mainly present in mammalian milk, which can be decomposed into glucose and galactose by lactase and then absorbed by the human body. But some people are lactose intolerant, namely, cannot break down and absorb lactose due to a lack of lactase. Previous research has found that lactose and its decomposition product of galactose and glucose in THz absorption spectrum has differences,^18^ so the THz-TDS can be applied to the determination of lactose content and degree of lactose tolerance of the human body. Most biomedical samples are only bioactive under water conditions, but water on all bands of THz wave has strong absorption effect; thus, in the case of water cut under test sample, the signal is difficult to be detected. Most current research is conducted on samples after dehydration, such as freeze-dried powders of some biological cells and bacteria.^19^

At present, most samples in the THz band test research rarely uses LiNbO_3_ crystal THz source of THz-TDS; most still use photoconductive antenna THz source of THz-TDS for biological macromolecules testing work.^20^

Related research teams have completed theoretical research and physical system construction on THz-TDS which uses LiNbO_3_ crystal as terahertz source and generates strong-field terahertz waves through optical rectification. As mentioned previously, ATR technology also has a lot of application research in terahertz biomedical detection, both of which are not new things. However, this paper for the first time combines ATR technology with LiNbO_3_ system to detect water-containing samples. On the one hand, we adjust the terahertz source to make the terahertz pulse power radiated as high as possible. On the other hand, we adjusted the detection method from the sample cell to the ATR prism to reduce the absorption of terahertz waves by water in the sample to be measured as much as possible. In this paper, we show the THz time-domain spectra drawn based on directly obtained data, the THz frequency-domain spectra, and the THz absorption spectra obtained by relevant calculation, as well as the plot of absorbance at 0.53 THz versus water content. In addition, we compared the test data of the sample cell in the LiNbO_3_ system and included the comparison results in the supplemental information.

## Results and discussion

### Results reliability discussion

**Figure 1:**
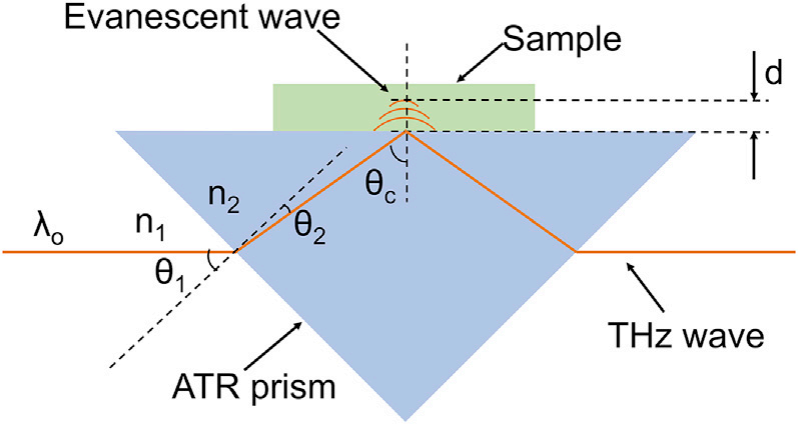
Schematic diagram of ATR prism sample

In the detection process, as shown in Figure 1, the high-resistance silicon prism used in this experiment has an incident angle *θ*_1_ of 45°, a refraction angle *θ*_2_ of 11.8°, and a total reflection angle *θc* of 56.8° according to the design of the prism and optical path. The relative refractive index can be described by the following Equation 1.

(Equation 1) $$n_{1}\;\sin\;\theta_{1}=n_{2}\;\sin\;\theta_{2}$$

where *n*_1_ is the refractive index of the incident medium (air) to the THz wave, *n*_2_ is the refractive index of the incident medium (high-resistance silicon) to the THz wave. The refractive index of the high-resistance silicon medium to the THz wave can be calculated as high as 3.46, and the penetration depth can be calculated by using the formula according to the nature of the evanescent wave. That is, the penetration distance of evanescent wave when the light intensity of the photophobic medium decays to 1/e, when total reflection occurs, and its expression is as follows in the Equation 2.^21^

(Equation 2) $$d=\frac{\lambda_{o}}{2\pi \sqrt{\left(\sin^{2}\theta_{c}-\frac{{n_{1}}^{2}}{{n_{2}}^{2}}\right)}}$$

where *d* is the penetration depth of the evanescent wave, *λo* is the wavelength of the incident light, *θc* is the total reflection angle of the beam, *n*_1_ is the refractive index of the THz wave in the medium with small refractive index optically, and *n*_2_ is the refractive index of the THz wave in the medium with large refractive index optically. The center frequency of incident THz wave *λo* in the formula can be obtained through the amplitude-frequency characteristic curve drawn from experimental data, which is 0.3 THz, and then combined with the relationship between frequency and wavelength, it can be calculated that *λo* is 1 mm. *θc* should be 56.8° according to the above prism and optical path design. *n*_1_, the refractive index of air to THz wave, is about 1 and *n*_2_, high-resistance silicon refractive index, is about 3.46. Substituting the above parameters into the Equation 2, it can be seen that when total reflection of THz wave occurs in the high-resistance silicon ATR prism, the penetration depth of THz wave is 0.203 mm. And the incident wavelength is in the same order of magnitude, which means that ATR spectrum can provide distance interface sub-millimeter or thinner layer of spectral information. It can be used for the detection of a small amount of biological samples. The thickness of the samples added in the experiment must be greater than this value to ensure that the evanescent wave can carry the information of the sample to be measured and finally ensure the repeatability of the experiment and the validity of the obtained data. In this research, the sample thickness was more than 0.5 mm, which met the experimental requirements.

### Detect results of α-lactose

**Figure 2:**
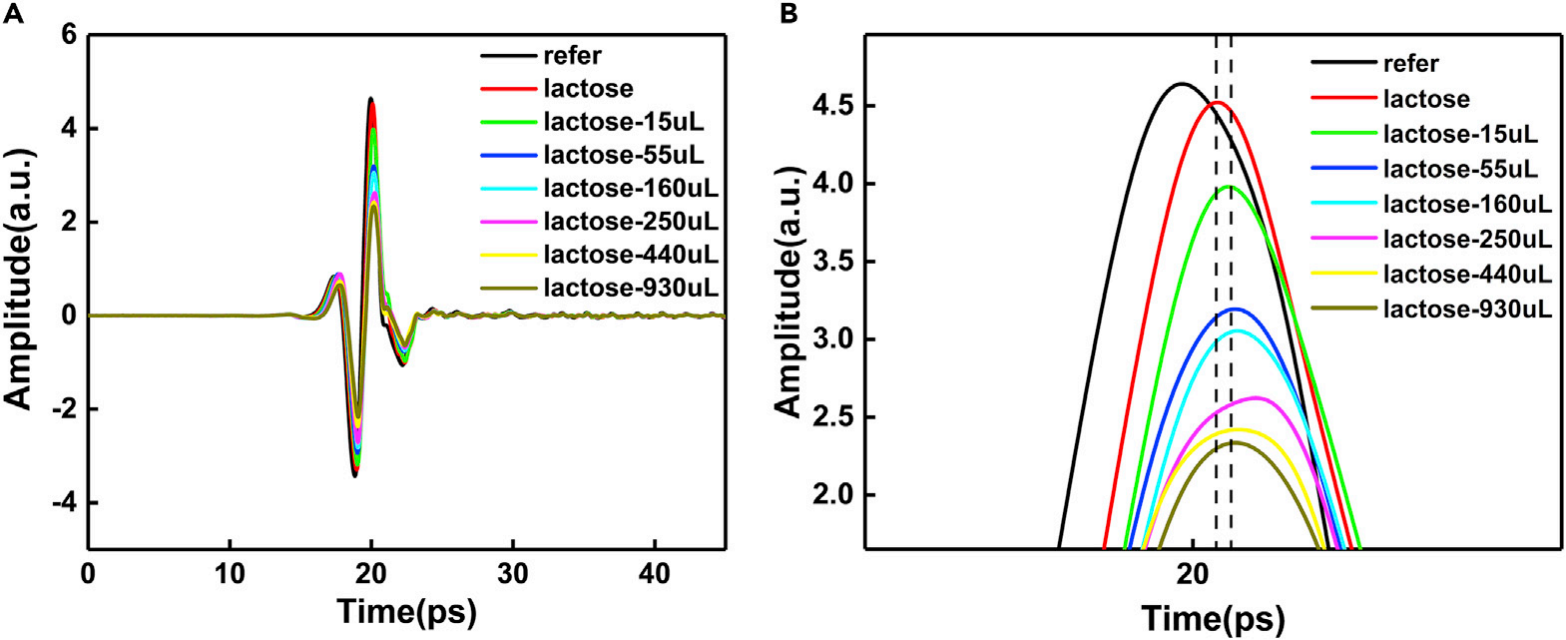
THz time-domain spectra of α-lactose samples with different water content (A) Complete time-domain spectral image. (B) Signal peak enlarged detail diagram.

On data processing, first of all, the obtained data are processed on the computer application software, and then the time-domain waveform figure is drawn. On the whole, it can be seen from Figure 2 that there is a difference between the peak and peak points of the time-domain waveform of the measured object and the reference waveform. Embodied in, peak appeared different degree of reduce, the time point of the peak appears with varying degrees of delay, and the peak will gradually decrease with the increase of the water amount. In terms of details, by observing the drawn time-domain waveform and the enlarged view of the peak, the following points can be seen. For the first point, compared with before and after adding samples to the ATR prism, it is found that the time point of the peak appears obviously delayed, and the amplitude decreases. The second point is that before and after adding water, the time point of the peak appears to be delayed again, but the degree of delay is small, and the amplitude decreases obviously. The third point is that with the increase of water quantity, the time point of the peak cannot be observed with obvious delay, and the decline degree of the amplitude increases with the increase of water quantity. These phenomena preliminarily indicate the following points: first, our sample test results are indeed different from the no-load scanning results of the system. Second, the signal of the sample before and after adding water is also different, indicating that the results of the test object in different states are indeed obtained. Third, after adding water, the position of the peak point of sample signal did not change significantly with the change of the amount of water added, indicating that the state of the medium through which the light carries information changes little and the sample is in a relatively stable and uniform state.

**Figure 3:**
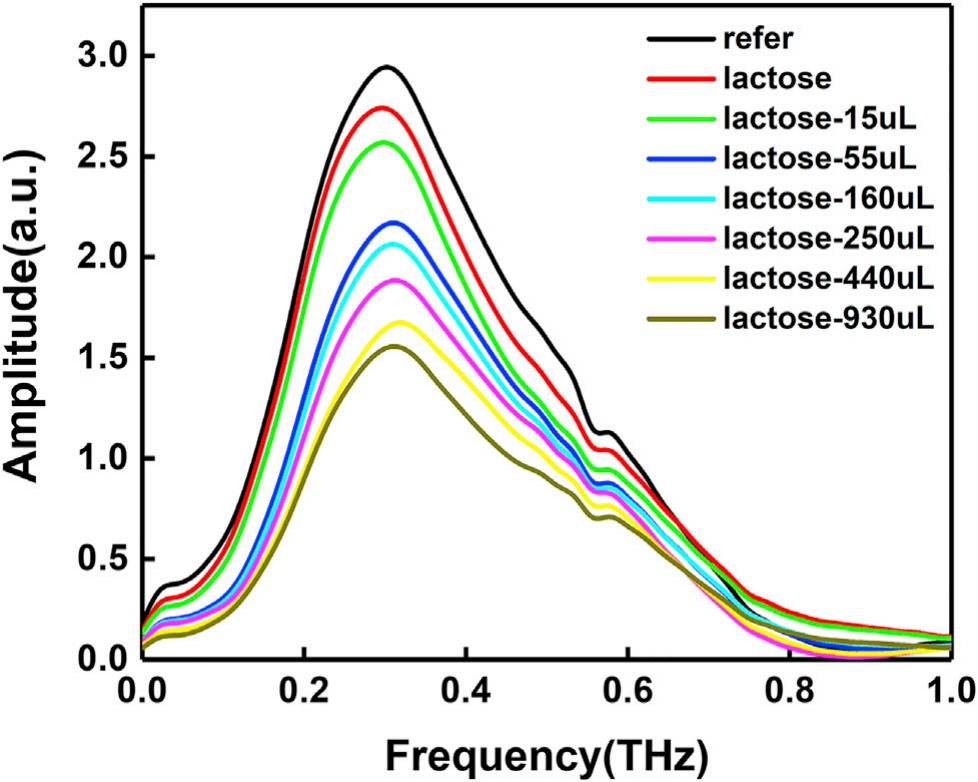
THz frequency-domain spectra of α-lactose samples with different water content

Secondly, according to the relationship between the time domain and frequency domain, we used Origin software to time domain Fourier transform frequency domain data and obtained the data and plot of the amplitude-frequency characteristics. It can be seen from Figure 3 that the peak frequency of the THz wave generated by the continuous ramp wavefront technique with LiNbO_3_ crystal as the source is about 0.3 THz. Before 0.7 THz, the amplitude-frequency characteristic curves of the ATR prism without load and after adding samples are basically the same, and with the increase of water added, the amplitude of the curve gradually decreased. After 0.7 THz, the curves show different degrees of crossover, indicating that the spectrum width of the system is about 0.1–0.7 THz.

**Figure 4:**
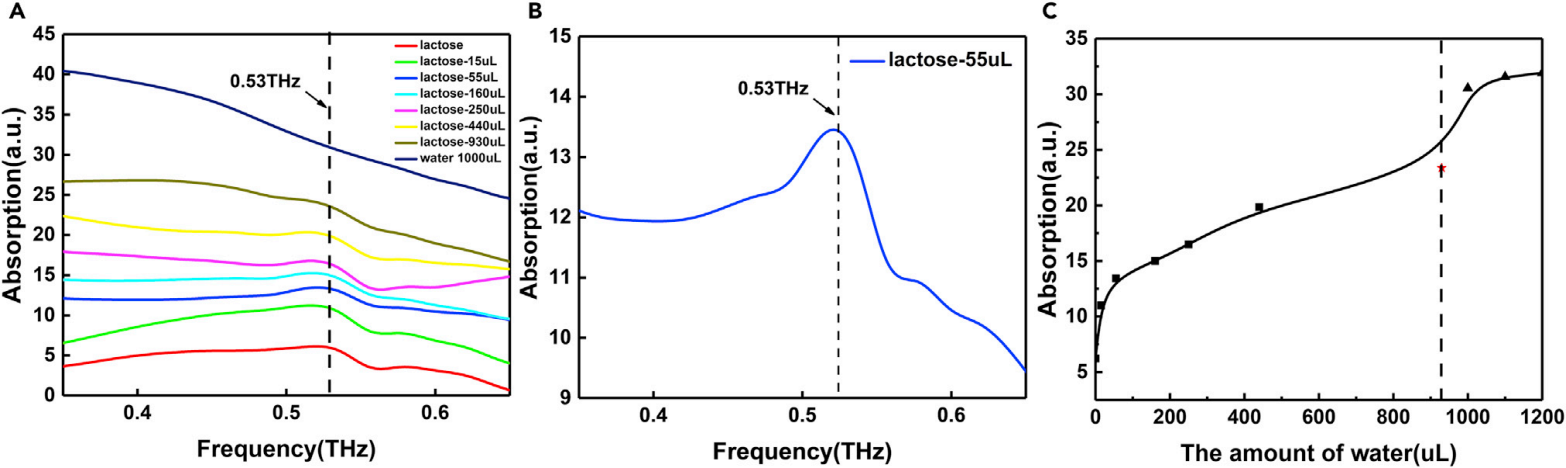
THz absorption spectra of α-lactose samples and the relationship between the absorption and water content (A) THz absorption spectra of α-lactose samples with different water content. (B) THz absorption spectra of α-lactose samples with 55 μL added water content. (C) Plot of absorption at 0.53 THz versus water content for samples tested with ATR prism.

Finally, according to the Beer-Lambert law, the absorption spectra are obtained by using the frequency domain data, and its expression is as follows in the Equation 3.

(Equation 3) $$A=-\log_{10}\left(\frac{I_{t}}{I_{o}}\right)$$

where *A* is the absorbance of the sample to be measured to the THz wave, *Io* is the intensity of the incident light, and *It* is the intensity of the outgoing light. The absorption spectra of the tested samples at different water content can be calculated and drawn by Origin software. The results are shown in Figure 4, where the absorption spectra of 55 μL of added water is shown separately so that the absorption peak at 0.53 THz can be observed more clearly. Moreover, as shown in Figures S2 and S3, we supplement and compare the test results of ATR and sample cell. Firstly, in the absorption spectra of the added water at 55 μL, it could be observed that the curve had an obvious absorption peak at 0.53 THz and the absorption peak existed at different water content. At the same time, with the increase of water content, the position of absorption curve moved up, indicating that the absorption degree of THz wave also increases. The above results indicated that the absorption peak of α-lactose at 0.53 THz was detected successfully by using the LiNbO_3_ system combined with ATR prism. And it was found that the absorption peak on the absorption spectrum became difficult to observe when the water was added to 930 μL. Owing to the limited experimental conditions, it was difficult to observe the effective absorption peak when the water content continued to increase. Therefore, our experimental results show that the detection limit of ATR prism on LiNbO_3_ system is temporarily 930 μL. In addition, when only 1000 μL of water was added to the ATR prism, it was found that there was no absorption peak at 0.53 THz. Moreover, the absorption peak at 0.53 THz disappeared, and the absorption coefficient began to be flat and did not rise when water was added to the sample to 1200 μL. These two points further verified that the absorption peak of α-lactose at 0.53 THz was indeed detected by the system. The absorption coefficients of the samples with different water contents at 0.53 THz were collected and then fitted by B-spline curves. The results are shown in Figure 4C, where the point of the red five-pointed star is the data point of the α-lactose sample with 930 μL of water added, and the dashed line represents the current test limit of this experiment. Compared with the sample cell shown in Figure S4, the test effect is significantly improved. And according to the weight of the α-lactose reagent, the amount of substance calculated is 0.29 mmol, which also shows that the system has been able to complete the sub-millimolar substance detection.

### Conclusions

In this research, the absorption peak of α-lactose at 0.53 THz at different water content was successfully measured by ATR method using THz-TDS of LiNbO_3_ crystal, which is a strong terahertz source based on optical rectification mode. In addition, compared with the sample cell test, this method can detect the absorption peak of sub-millimolar samples with a water content of 930 μL, which significantly improves the sensitivity and detection limit of water-containing samples, that is, it is more sensitive to the change of water content and can detect samples with a higher water content. It provided a feasible way to solve the problem that water has strong absorption effect on THz wave in the current research field. However, the current test spectrum is relatively narrow, and the spectrum width of 0.1–0.7 THz is still limited for testing about most biological macromolecules whose absorption peaks are above 1 THz. At present, the use of ATR coupled with THz-TDS, which can generate strong field THz wave, to quantitatively test aqueous samples is still in its infancy. There is still a certain gap in the test of dilute solution in the real sense, and the combination of ATR with other methods or the improvement of ATR can be considered in the experimental design to further improve the sensitivity and detection limit of aqueous samples.

### Limitations of the study

According to the absorption spectra, the absorption peaks of the results obtained in this experiment are not obvious compared with the results of the sample cell test when the α-lactose powder is also tested. The spectral width of the experimental system is only 0.1–0.7 THz in the sight of THz frequency-domain spectra, which will limit its further application. So how to further broaden the spectrum and enhance the feature peak is the focus and difficulty in this study.

## STAR★Methods

### Key resources table

**Table:** 

REAGENT or RESOURCE	SOURCE	IDENTIFIER
**Chemicals, peptides, and recombinant proteins**		

α-Lactose monohydrate	Aladdin	CAS#5989-81-1

**Software and algorithms**		

Origin 2017	Origin Lab	https://www.originlab.com/

### Resource availability

#### Lead contact

Further information and requests for resources and reagents should be directed to and will be fulfilled by the lead contact, Wei Shi (swshi@mail.xaut.edu.cn).

#### Materials availability

This study did not generate new unique materials.

### Experimental model and subject details

Our study does not use experimental models typical in the life sciences.

### Method details

#### Sample preparation

There are two isomers of lactose, α-lactose and β-lactose, among which α-lactose is easy to form α-lactose hydrate with a molecule of crystal water, while Milk mainly contains α-lactose. Therefore, this work mainly took α-lactose hydrate as the starting point. The α-lactose powder used in the experiment was the analytical pure α-lactose reagent from Aladdin reagent, and the water used was deionized water. An electronic analytical balance with a precision of 0.01 mg was used, and a pipetting gun with a range of 10-100 μL and an increment of 1 μL was used. During the test, an electronic analytical balance was used to weigh 0.105g α-lactose sample. The sample to be tested was directly placed on the ATR prism, and then the water dissolved reagent was quantitatively added through the pipette gun for testing. The water added was 15 μL, 55 μL, 160 μL, 250 μL, 440 μL and 930 μL respectively. At the same time, a sealing ring was placed on the surface of the ATR prism to ensure that the water would not spread around.

#### System building

To test the samples, it is necessary to use the THz-TDS for detection. Our laboratory’s system uses optical rectification method and LiNbO_3_ crystal as the excitation source to generate THz wave. Optical path diagram of THz-TDS is shown in Figure S5. MaiTai XF-1 titanium Sapphire femtosecond laser oscillator produced by spectra-physics was mainly used as the seed source. The laser with center wavelength of 800 nm, repetition rate of 80 MHz, pulse width of 80fs and output power of 0.6W was generated. After amplification by Spitfire ACE-100F laser amplifier produced by Spectra-Physics, the laser beam with pulse width of about 100fs and power of 4W is generated. After entering THz-TDS, the laser beam is divided into two beams, the detection channel and the pump channel, through the beam splitter. Among them, the laser in the pump path will first pass through a shining grating (shining Angle 28.7°, shining wavelength 800 nm, cutting density 1200/mm) to generate a fixed wave-front tilt Angle to meet the phase matching conditions in the process of LiNbO_3_ crystal rectifying. After passing through a specific cutting Angle of 63° LiNbO_3_ crystal, the sample to be tested will generate a THz wave with an average power of 500 μW through the optical difference frequency, and then pass through the focusing optical path between the second and third surfaces of four off-axis objective lenses, and place it on a high resistance silicon ATR prism with resistance greater than 10,000 ω/cm. Before placing the ATR prism, we measured the spot diameter using a pyroelectric array camera and determined the diameter of the THz spot to be 2.5 mm. The ATR prism is isosceles right-angle prism, as shown in Figure S6, the cross section of the triangle to 48 mm size, 34 mm, 34 mm, thickness of 21 mm, as shown in the inversion using 3D printing fixed shell, will need to test the samples placed or smear on the surface, the THz wave in the high resistance silicon prism placed on the surface of the sample total reflection occurs, when total reflection occurs, the THz wave is not absolutely reflected back to the ATR prism at the interface, but penetrates into the object to be measured at a depth of about one wavelength, flows through the distance of wavelength magnitude along the interface, and then returns to the ATR prism, and is emitted along the reflected light direction. This process completes the detection of the sample and carries the information of the sample through the fourth off-axis parabolic mirror to the ITO membrane; The laser in the detection path will pass through a translation table controlled by the instrument, where a pair of mutually perpendicular mirrors are placed on the translation table to form a time delay system. The purpose is to change the optical path of the detection path through translation to make it consistent with the optical path of the pump path, and then reach the ITO film through the mirror. Pump and detecting road of THz wave and the laser overlap on the ITO film, and at the same time through ZnTe crystal electro-optic sampling, the way is the THz wave irradiation to ZnTe crystal due to general Kerr effect change detection way of laser polarization, such changes are caused by THz wave produced by the electric field intensity, so also can detect THz wave waveform, Then the linearly polarized light is changed into circularly polarized light through a quarter-wave plate, and then the Wollaston prism is used to separate O light and E light with mutually perpendicular polarization, and then the signal is detected by a differential equilibrium detector, and the equivalent time sampling principle is used. By adjusting the time delay system by controlling the translation stage, the optical path of the detection path is changed so that the optical path difference between the detection path and the pump path is changed. The time-domain waveform data of THz pulse is measured and obtained by using lock-in amplifier and computer system.

#### Sample testing

In this process, firstly we need to purge dry air throughout the system to ensure that the humidity of the test environment is not higher than 3%. Secondly, we need to test air drying system under no-load signal-to-noise ratio, through optimizing the debugging system makes the SNR can be greater than 10,000. The SNR can be calculated by the ratio of the peak-to-peak value of the scanned signal to the root-mean-square of the first 30 data of the scanned signal while in the first 30 data of the scanned signal the peak-to-peak value of the scanned signal has not yet appeared, so the calculated root-mean-square value is regarded as the noise size and the peak-to-peak value is regarded as the signal size. Thirdly, as shown in Figure S1, we need to test the ATR prism no-load state as a reference signal, then add the sample powder for testing. In the subsequent test, we use a pipette gun to quantitatively add deionized water to mix the samples. After the samples are evenly mixed, a test is completed to obtain the corresponding test data results, and then the quantitative water test is continued to obtain multiple groups of data.

### Quantification and statistical analysis

There is no statistical analysis in this paper.

### Additional resources

We have no relevant resources.

## Data Availability

• All data reported in this paper will be shared by the lead contact upon request. • This paper does not report original code. • Any additional information required to reanalyze the data reported in this paper is available from the lead contact upon request.
