# Supplementary material for: Quantitative detection of THz-ATR spectra of aqueous samples under strong-field terahertz wave
Source: iScience. 2022 Dec 24;26(1):105871. doi: 10.1016/j.isci.2022.105871 (PMC9826933; doi:10.1016/j.isci.2022.105871)
Supplement: Document S1. Figures S1–S6 [file mmc1.pdf]

iScience, Volume *26*

Supplemental information

Quantitative detection of THz-ATR spectra of aqueous samples under strong-field terahertz wave

Wei Shi, Chunhui Li, Haiqing Wang, Zhiquan Wang, and Lei Yang

iScience

Supplemental information

This PDF file includes

Figure S1. Diagram of steps and sample states when adding samples to the ATR prism, related to STAR Methods.

Figure S2. THz absorption spectra of a α-lactose sample with a water content of 10μL on the sample cell, related to Figure 4(b).

Figure S3. THz absorption spectra of a α-lactose sample with a water content of 55μL on the ATR prism, related to Figure 4(b).

Figure S4. Plot of absorption coefficient versus water content at 0.53THz for samples tested using ATR prism and sample cell, related to Figure 4(c).

Figure S5. Schematic diagram of THz-TDS, related to STAR Methods.

Figure S6. Stereoscopic diagram of ATR prism sample test, related to STAR Methods.

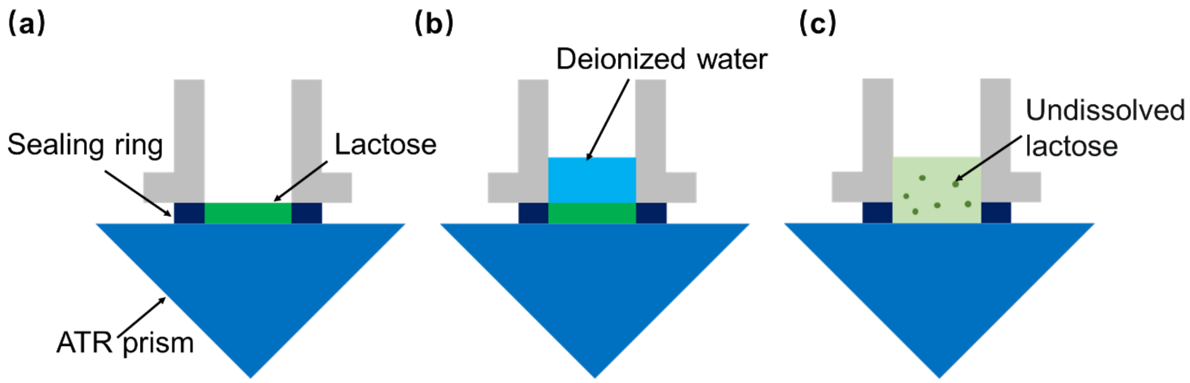

Figure S1. Diagram of steps and sample states when adding samples to the ATR prism, related to STAR Methods. When placing the sample, as shown in Figure S1, first lay the α-lactose powder on the surface of the ATR prism, and then add water after testing the powder. Since the solubility of α-lactose is 0.216 g/mL at room temperature, the sample will not be completely dissolved at the beginning of adding water, but in the state of suspension as shown in Figure 1c. Because the quantity of samples to be tested is small, we use air blowing as a stirring device to make the samples to be tested in a relatively uniform state, the specific operation is to gently blow the liquid surface with air to mix evenly. The sealing ring is made by twisting Teflon thread sealing tape and then glued to the shell of the prism.

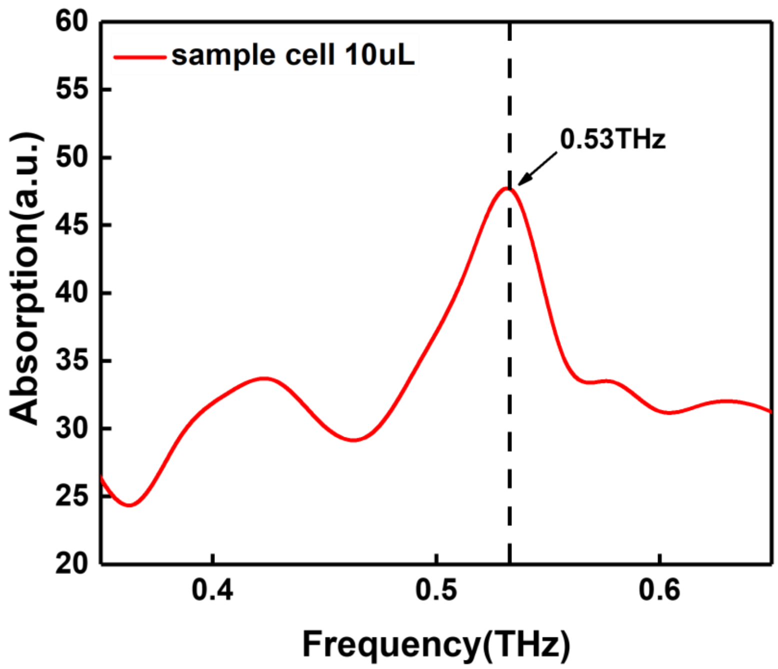

Figure S2. THz absorption spectra of a α-lactose sample with a water content of 10μL on the sample cell, related to Figure 4(b).

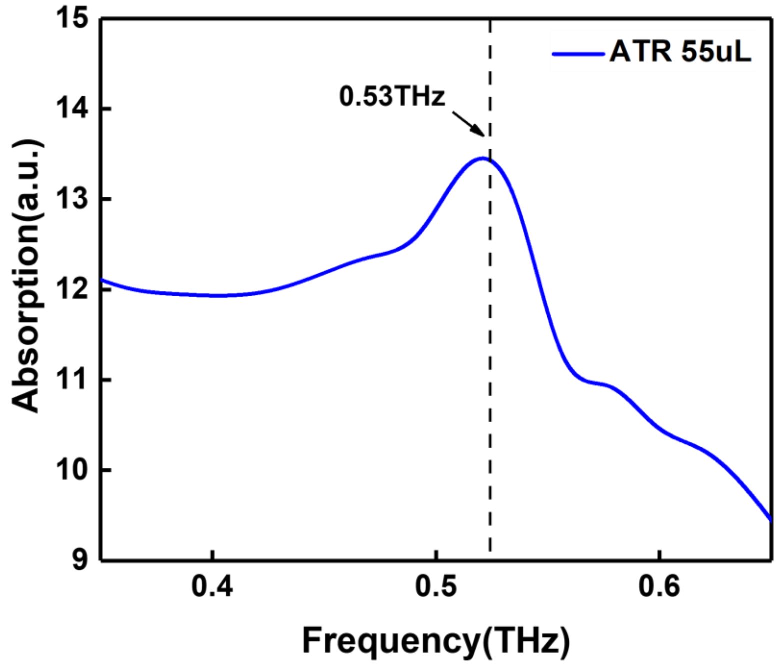

Figure S3. THz absorption spectra of a α-lactose sample with a water content of 55μL on the ATR prism, related to Figure 4(b).

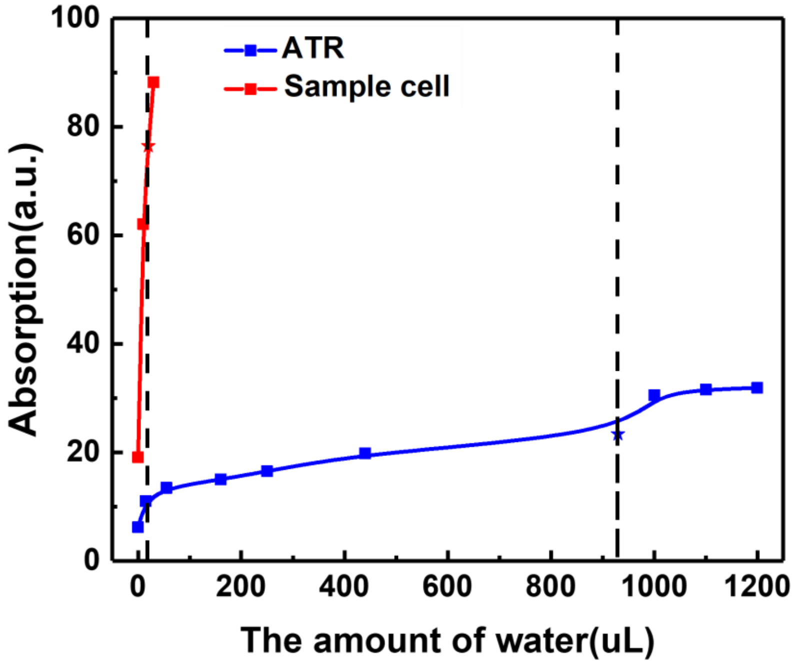

Figure S4. Plot of absorption coefficient versus water content at 0.53THz for samples tested using ATR prism and sample cell, related to Figure 4(c). As shown in Figure S2 and Figure S3, the absorption peak of α-lactose at 0.53THz can be tested in either the sample cell or ATR prism, the sharpness of the absorption peak of the ATR prism detection

result is lower than that of the sample cell in the map drawn according to the test results. However, as shown in Figure S4, the absorption peak of α-lactose at 0.53THz could not be detected when the water added to the sample cell reached 20μL, while the water added to the ATR prism in this experiment could reach 930μL. The results of the sample cell test have major limitations for most studies that want to test biological samples under conditions of retention of biological activity. At the same time, we have the following problems during the sample cell test: first, the operation of adding samples to the sample cell is relatively complicated; second, how to fix the sample cell and ensure that it is in the same position as far as possible in each test.

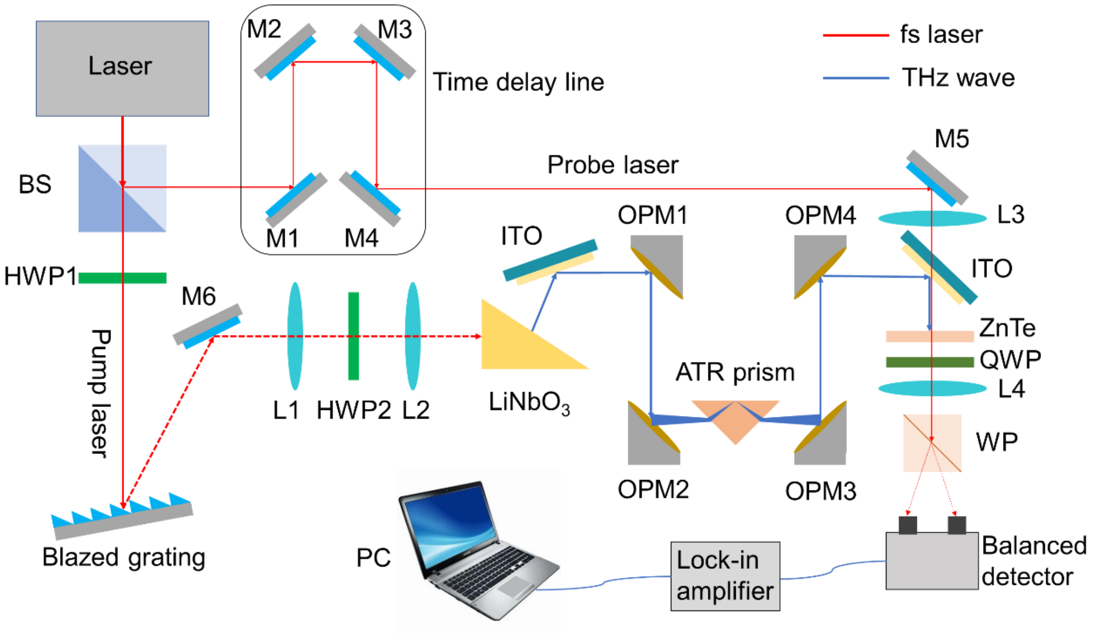

Figure S5. Schematic diagram of THz-TDS, related to STAR Methods. BS: beam splitter; HWP: half wave plate; OPM: off-axis parabolic mirror; P: polarizer; M: mirror; ITO: indium tin oxide; QWP: quarter wave plate; PC: personal computer; WP: Wollaston prism; L: lens.

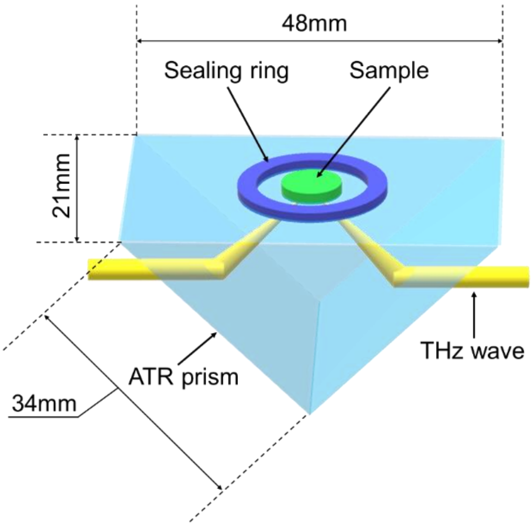

Figure S6. Stereoscopic diagram of ATR prism sample test, related to STAR Methods.
